# Extraction of Teeth

**Published:** 1875-01

**Authors:** 


### ARTICLE II.

### *Extraction of Teeth.*

BY DR. EATON.

Read before the California State Dental Association.

The subject selected, "The Extraction of Teeth," is one that has not been written on or discussed by this Society in former years, perhaps because every member was supposed to know all about the *modus operandi*, and partly for the lack of anything new or novel, either in the way of instruments or methods of using them, for some time past. Notwithstanding that to be able to extract understandingly, requires an accurate knowledge of the shapes and peculiarities of the different teeth, as well as the surrounding tissues. It has not been many years since the common practice was to remove all teeth that were troublesome, from whatever cause. At the present time, a dentist would not be considered worthy of the name should he consent to perform such an operation, if there was a reasonable probability of saving the offending member by a course of treatment, or until the patient was fully informed of the chances, and still insisted on its removal. In the treatment of diseased and aching teeth, there has been wonderful advances made in the right direction, especially on this coast, since the State Dental Association has been organized, and there is still room for improvement. Those that are farthest advanced in the profession are the ones who are most anxious to learn, and most willing to impart their knowledge to others. This is as it should be, and I am sure there is not a member of this Association that has not been benefited and repaid many times over for all the inconveniences and loss of time sustained by attending. One great reason why more diseased teeth are not successfully treated in the country is that a large number of patients have been told by traveling quacks, that after a tooth once aches the quicker it is extracted the better; such an one, if persuaded to allow you to make an application to destroy a nerve, for instance with instructions to return in twenty-four hours, many times will not be seen again for weeks; and perhaps, would not come then if the tooth had not become sore and troublesome again, and they want it filled as you informed them it probably could be when you first saw the case. This time you are not so hopeful of success, as it may require several dressings before being in a condition to fill, and the result many times is, that the tooth is finally lost more through the patient's fault than yours; but you have all the blame to bear, and he is only strengthened in his opinion, held from the first, and would be indignant should the fee be more than for the simple extraction. Before anything is done, every case presented should be carefully examined, in order to ascertain as much as possible the circumstances and surroundings that might affect the operation, so that a correct conclusion may be formed of the number and inclination of the roots, and the probable amount of force that will be required for their removal; also satisfy yourself which tooth is the real cause of the trouble, for many times the patient will locate the pain in the wrong one, that perhaps stands in contact, and may be affected to some extent, but which will cease to trouble on the removal of its diseased neighbor. The manner of operating is also of importance, as well as the selection of a proper forcep. I shall always remember the frantic efforts of an old dentist in the East, some ten years ago, in whose office I had just been employed as assistant: A gentleman took a seat in the chair, and wished a superior molar extracted ; the proprietor selected a forcep, grasped the tooth, shut his eyes, and then began such a series of jerks and pulls as I never saw equalled, which continued until he was tired completely out, when he asked me to take a pull at it, as he had lost his grip entirely. Having come directly from an office where I had had the benefit of five years' instruction with a very cool and sure operator, had no trouble in breaking up the attachment by giving it the usual outward and inward motion. Much mischief is often done by such nervous operators, and accidents of a serious character occur, such as fractures of the alveolus and sometimes the maxilla causing permanent deformity. A description of forceps suitable for extracting the various sizes and shapes of teeth, would be out of place here, a great deal depending on being accustomed to an instrument, and one that would come handy to me might be awkward to another. The great point is to have a good variety of different shapes and sizes so that no trouble will be experienced in selecting one that will fit nicely any case that may present itself; then, after lancing the gum well, in such cases as require it, secure a deep hold, and don't get excited. Prof. Taft says: "A very good criterion is that the eye should critically follow, and the mind attentively comprehend every movement of the hand and instrument." The forcep is considered by most operators the best and safest; but there are many cases where elevators, screws, hooks, etc., are of great assistance. The key is still used by some good operators ; but having had no experience except as a patient, I am not prepared to say anything in its favor. To be able to extract well is a great help in securing patronage in other branches of our profession ; for although it is usually one of the easiest labors we have to perform, it is dreaded more than any other by our patients. I have known at least one dentist, who though no gentleman, and having but few warm friends, was sought for from far and near for his known skill in extracting. The indications for extracting are, in many cases, very plain to be seen at a glance. Temporary teeth that retain their place until the corresponding permanent ones appear, should always be removed; also temporary teeth that are in such a diseased condition as to do more harm than good if retained. I notice that Isaiah Forbes, D. D. S., in a paper on irregularities, advocates extracting all deciduous teeth that are presented which are badly decayed and aching, contending that the extraction of such teeth cannot possibly do any harm; but on the contrary, even though by an application we could alleviate the pain, their retention might injure the healthy growth of the permanent teeth. He says: "There is nothing so difficult to change as the impressions and prejudices of our early education. The more I have studied and reflected on this branch of the subject, the more have I become satisfied that the doctrine as taught in our dental text book cannot be correct. I also have the proud satisfaction of learning that Mr. Tomes, in his histological invetigations has come to the same conclusion." He goes on to illustrate by mentioning a case in his own family, and in a long argument makes a good showing in favor of his theory. This calls out in opposition, a long article from H. L. Sage, D. D. S.; which theory is best, time and investigation will no doubt bring forth. I have followed the method laid down in our text books, and endeavored to save the deciduous teeth as long as they could be retained, providing they are not in the way of the permanent ones. The matter of trying to save the first, or six year old molars, is another point of controversy on which much has been said both for and against. It seems to me that the Great Architect of the Universe must have placed them in their position, to be retained and made useful, as well as the twenty-eight remaining ones. It certainly is a serious matter to the little folks when they have to submit to their extraction—all painful and diseased roots should be extracted. An incurable abscess is generally sufficient cause for extraction ; or an abscess threatening to point on the outside of the face ; also teeth that are so sore as to cause a continued avoidance of that side of the mouth in eating, if they cannot be cured should be removed.

Extracting for irregularities is sometimes necessary, but no rule can be laid down to govern such cases ; great care and good judgment is requisite. In extracting for the insertion of artificial dentures it is often difficult to decide for the best, as to how many to remove, and how many to leave, and when our mind is once made up, it is often a hard matter to bring our patient to the same way of thinking.

In cases where a large number of teeth are badly decayed, so that the expense of saving them would be double or treble that of plate, we are often obliged to extract against our wishes. To quote the words of another : " How far we can go in urging the preservation of such teeth ; how much we can do to strengthen faith in the permanence of fillings ; how much we may dictate to our patients as to what they can or cannot afford, and how much charity we shall bestow to induce them to undertake the best plan, are questions which each must decide as best he can in each case ; only let me plead that in each case the decision be made with a sincere desire to promote the best interests of the patient, and not on the basis of our own self interest.

